# Nucleic Acid Amplification Testing for Human Immunodeficiency Virus, Hepatitis B Virus, and Hepatitis C Virus in Blood Donors at a Tertiary Care Hospital Blood Bank

**DOI:** 10.7759/cureus.77571

**Published:** 2025-01-17

**Authors:** Shaheen Kouser, Hira Qadir, Maira Ahmad, Hira Tahir, Mehwish Sajjad, Fakhrunnisa Khan, Ghazia Shamim

**Affiliations:** 1 Hematology, Dow University of Health Sciences, Dow International Medical College, Karachi, PAK

**Keywords:** hepatitis b, hepatitis c, hiv, nucleic acid amplification techniques, transfusion transmitted infections

## Abstract

Objective

The objective was to assess the efficacy of nucleic acid amplification testing (NAAT) in conjunction with serology for detecting hepatitis B virus (HBV), hepatitis C virus (HCV), and human immunodeficiency virus (HIV) in donor blood products.

Methodology

This retrospective cross-sectional study aimed to evaluate the use of NAAT and viral serology in screening donor blood products for HBV, HCV, and HIV. The study was conducted at the Dr Ishrat-ul-Ebad Khan Khan Institute of Blood Diseases at Dow University of Health Sciences in Karachi, Pakistan, from April 2018 to May 2022. All blood products, after preparation, were screened for HBV, HCV, and HIV types I and II using electrochemiluminescence immunoassay on the Roche cobas e 601 analyzer (Roche, Basel, Switzerland). For further confirmation, blood products with negative viral serology underwent NAAT using the cobas TaqScreen MPX test version 2.0 (Roche) on the cobas s 201 system (Roche) to detect the window period of replicating viruses. Simple calculations were performed to determine the NAAT yield and yield rate for each virus, as well as the total number of viruses in seronegative donors.

Results

A total of 46,455 donors visited the Dr Ishrat-ul-Ebad Khan Khan Institute of Blood Diseases for blood donation. Of these, 6.97% (n = 3,240) tested positive for HIV, HBV, or HCV during serology screening, making them ineligible for donation. The remaining 93.02% (n = 43,215) of seronegative donors underwent NAAT to detect infections during the window period. NAAT revealed a reactivity of 0.044% (n = 19) for HBV, 0.009% (n = 4) for HCV, and 0.00% (n = 0) for HIV. The total NAAT yield rate for HBV was 1 in 2,252, and for HCV, it was 1 in 11,111. The overall NAAT yield rate for all viruses was 1 in 1,886 (0.053%) donor blood products.

Conclusions

These findings highlight the effectiveness of NAAT in identifying blood-borne infections during the window period. Consequently, routine NAAT screening for all seronegative blood donors is a crucial step that can reduce the burden of transfusion-transmitted infections and enhance the safety of blood products, especially in underdeveloped countries like Pakistan.

## Introduction

During the 1980s, human immunodeficiency virus (HIV), followed by hepatitis B virus (HBV) and hepatitis C virus (HCV), were becoming prevalent worldwide as they were being found in recipients after transfusions [1]. To target this issue, many developed countries started to use nucleic acid amplification testing (NAAT) as a screening method to detect viral DNA, leading to NAAT now being a standard measure taken before giving blood. Overall, NAAT has severely reduced the risk of transmissibility of blood-borne diseases in these countries by serving as a preventative measure in blood donation [2].

Although many developed countries have accepted this procedure, many of their transfusion centers still do not include NAAT screening to ensure the safety of blood products. This is the case in Pakistan, where very few transfusion centers have made the decision to use NAAT as it is not a mandatory requirement [3]. There are still barriers to achieving widespread use of NAAT, as Pakistan does not have the necessary resources to fund regular NAAT screening, and it is still considered expensive. Shortages of electricity, expensive equipment, and testing supplies, and negligent surveillance have led to many blood banks straying away from using NAAT. This puts both the recipient and donor at risk, as donors may not know they have contracted the disease until symptoms arise [4]. One study conducted in Pakistan reported that out of 54,438 seronegative blood donors screened using NAAT, 23 donors were reactive for HBV and four were reactive for HCV [3]. A similar study, using a chemiluminescence immunoassay and NAAT, found HCV to have the highest prevalence at 1.7%, followed by HBV at 1.5%, and 0.07% for HIV [5]. Another study revealed that most donors are usually first-timers, which could be a causative factor for the higher prevalence of infection since they have never been screened before. In fact, Pakistan has the second highest prevalence of HCV in the world, emphasizing the urgent need for the introduction of screening for blood products [6]. As of 2020, the overall prevalence of HBV was 1.6%, while HCV had a prevalence of 7.5%, with 545,000 incident cases per year. Meanwhile, in 2023, HIV had a prevalence rate of 0.2% for individuals aged 15-49 years old [7]. In other words, 290,000 individuals aged 15 and over were infected with HIV. Additionally, 8,300 children aged 14 and under were also infected with HIV.

Unfortunately, Pakistan has a high prevalence of all three blood-borne diseases, as HIV patients are at a greater risk of contracting HBV and HCV. While nationwide prevalence rates of coinfection have not yet been published, especially using NAAT testing, a few studies have provided some insight into this issue. For example, Masroor et al. used enzyme-linked immunosorbent assay and RT-PCR to screen 650 HIV patients for HBV and HCV [8]. Of the 650 patients, 78 (12%) were found to have hepatitis, with 63 (80.77%) testing positive for HBV and 15 (19.3%) testing positive for HCV. Another study conducted among 108,598 blood donors found 103 donors (0.09%) to have coinfection [9]. Overall, five (0.004%) donors were coinfected with HIV and HBV; two (0.001%) were coinfected with HIV and HCV; 94 (0.084%) were coinfected with HBV and HCV; and two (0.001%) were infected with all three viruses. From 2004 to 2023, these studies show that despite the passage of time, the prevalence of hepatitis coinfections in HIV patients still continues to pose a threat, while the necessary actions to address the issue have yet to be taken.

Overall, all articles covering this issue echo the same message of NAAT screening and confirmatory testing becoming a necessary part of blood donation to lower the frequency of HBV, HCV, and HIV transmissibility through blood products [10]. Thus, we hope to create awareness and educate on the standard practice of using NAAT to screen blood and for the proper management of public funds to support this method to lower the infectious burden on Pakistan’s health infrastructure.

## Materials and methods

This was a retrospective cross-sectional study with data gathered over five years, from April 2018 to May 2022. After approval for exemption from the Institutional Review Board (IRB) at Dow University of Health Sciences was issued on April 21, 2022 (IRB reference number 2509), the study commenced. Using a simple random sampling technique, data was obtained from the Dow Diagnostic Research and Reference Laboratory, Hematology Department at the Dr Ishrat-ul-Ebad Khan Khan Institute of Blood Diseases under Dow University of Health Sciences from 46,455 donors. The blood bank within the department used routine screening via the two-step method of serology and NAAT to screen blood products.

Serology was first used to screen the donor blood for HIV, HBV, and HCV to exclude any blood donor products found to be positive. Blood donor products that were found to be negative on serology were then run through NAAT for confirmation of negative results for HIV, HBV, and HCV. Personal data provided by donors remained anonymous, and only data on age and gender was included in the study. Inclusion criteria included both genders aged 18-50 who were seronegative for HBV, HCV, and HIV and showed no symptoms of sickness before donation. Exclusion criteria included donors younger than 18 and older than 50 years and those found positive on serology.

After informed and written consent was given, blood was taken from patients in a standard manner and prepared for screening. All blood products after preparation underwent screening for HBV, HCV, and HIV types I and II, through an electrochemiluminescence immunoassay (ECLIA) by Roche Cobas e 601 analyzer (Roche, Basel, Switzerland). Blood products that were found reactive on serology with ECLIA were excluded, while blood products that were not reactive were included. The seronegative blood products were then tested for further confirmation using NAAT (RT-PCR) by cobas TaqScreen MPX test version 2.0 (Roche), used on the cobas s 201 system to detect the window period of replicating HBV, HCV, and HIV viruses. Seronegative blood products were first put into 6-unit mini-pools on NAAT testing. If any of the six samples were found positive for HIV, HBV, and/or HCV, the pool was reopened and testing was done similar to that of individual donor NAAT testing to check for which of the six samples were positive for which viruses. This reopened and retested pool was known as a resolution pool. Once found, that particular blood donor product was safely discarded.

All data was organized in Microsoft Excel (Microsoft Corporation, Redmond, WA, USA) and categorized according to patients’ sex, year of collection, reactivity and non-reactivity on serology, and reactivity and non-reactivity on NAAT. The data was analyzed using simple calculations to check the frequency of reactive cases on NAAT for each virus and for the total of all three viruses from 2018 to 2022. We calculated NAAT yield and NAAT yield rate by following the basic written formula by Ali et al. [5], which were written following the detailed descriptions originally provided by Busch et al. [11] on these calculations. The formulas for NAAT yield and NAAT yield rate are provided by Busch et al. [11] and simplified by Ali et al. [5]. All formulas used for calculations are as follows: NAAT yield = (reactive donors on NAAT/total seronegative donors screened with NAAT) × 100 [5,11]. NAAT yield rate per million donors = (NAAT yield/total seronegative donors screened with NAAT) × 10^6^ [5,11].

## Results

A total of 46,455 blood donors were enrolled in the Dr Ishrat-ul-Ebad Khan Khan Institute of Blood Diseases from April 2018 to May 2022. The mean age for blood donors was years old. Out of 46,455 blood donors, 99.9% (n = 46,400) were male, while 0.1% (n = 55) were female. Around 90% (n = 41,809) of donors were replacement donors (the recipient's family members who donated blood).

After obtaining and preparing the blood products, these blood products were checked for reactivity on viral serology. Those deemed reactive on serology were referred to as “seropositive” and excluded from further screening for HBV, HCV, and HIV on NAAT. In total, seropositive donors were 3,240 in number (6.97%). After the exclusion of seropositive donors, a total of 43,215 (93.02%) seronegative (those who were not reactive to the three viruses on serology) donors were again screened on NAAT for the detection of infection of each virus’s window period. 

Out of 43,215 seronegative donors screened by NAAT, 19 donors were found to be positive for HBV with a NAAT yield of 0.044%. Meanwhile, four out of 43,215 seronegative donors were found positive for HCV infection over the five years, giving a NAAT yield of 0.009%. In contrast, no donor was found positive for HIV on NAAT during this period. Table 1 summarizes the comparison of donor reactivity on serology and NAAT over the five years. Results for HIV reactivity were deemed not necessary to be included in the table as there was no reactivity on NAAT.

**Table 1:** Results for blood donor products on serology and NAAT HBV, hepatitis B virus; HCV, hepatitis C virus; NAAT, nucleic acid amplification testing

Year	Donors	Reactive donors on serology	Non-reactive donors on serology	Resolution pool	Non-reactive donors on NAAT	Reactive donors on NAAT (HBV)	Reactive donors on NAAT (HCV)
2018	6,632	544	6,088	16	6,085	3	0
2019	11,105	801	10,304	24	10,296	7	1
2020	10,406	672	9,734	28	9,728	6	0
2021	12,680	836	11,844	29	11,841	0	3
2022	5,632	387	5,245	8	4,784	3	0
Total (5 years)	46,455	3,240	43,215	105	42,734	19	4

Yield rates for HBV, HCV, and HIV were calculated to find the rate of disease per one million donors. HBV was found to have the highest NAAT yield rate of 1 in 2,252 donors (0.044%). This was followed by the HCV which had a NAAT yield rate of 1 in 11,111 donors (0.009%). Overall, the total NAAT yield calculated was found to be 0.053% with a yield rate of HBV, HCV, and HIV occurring in 1 out of 1,886 blood donors. To allow for a better understanding, Table 2 summarizes these findings for NAAT yield and yield rate for each virus tested in seronegative donors.

**Table 2:** NAAT yield and yield rate for HBV, HCV, and HIV The formulas used for calculations were derived from references [5] and [11]. HBV, hepatitis B virus; HCV, hepatitis C virus; HIV, human immunodeficiency virus; NAAT, nucleic acid amplification testing

Years (total)	Virus	Total seronegative donors	Reactive seronegative donors found on NAAT	NAAT yield result	Yield rate result per million blood donors
2018-2022 (5)	HBV	43,215	19	0.044%	1 out of 2,252
2018-2022 (5)	HCV	43,215	4	0.009%	1 out of 11,111
2018-2022 (5)	HIV	43,215	0	0.000%	Nil
2018-2022 (5)	Total	43,215	23	0.053%	1 out of 1,886

## Discussion

Pakistan has the second-highest global burden of HCV, with a nationwide prevalence of 4.8% [12]. From 2015 to 2019, HCV-related deaths have increased by 5%, while those for HBV have increased by 8%. HCV and HBV transmission continues to increase due to socioeconomic instability, the inability to provide adequate means of hepatitis prevention, and insufficient availability of treatment [13]. The national and provincial hepatitis control program within Pakistan began providing antiretroviral treatment to patients with chronic HCV in 2005, but studies show that despite 150,000-160,000 patients receiving treatment, these numbers are unlikely to eradicate HCV in Pakistan [14]. Moreover, Qureshi et al. reported that only 74% of Pakistanis have been vaccinated against HBV [6]. According to a study done by Ochani et al., 1,634,614 patients were registered at various hepatitis clinics, yet only 278,308 patients were treated [12]. The lack of affordability for drugs to treat hepatitis combined with inadequate screening leads to many undiagnosed cases of HCV, resulting in complications such as liver cirrhosis and hepatocellular carcinoma [15]. It was shown that proper government or private funding for HCV elimination could reduce overall hepatitis morbidity and the burden on the Pakistani healthcare system [16].

Paid blood donations in Pakistan are common and often attract individuals who need money to pay for injectable drugs, which contributes to the exposure of HCV and HBV within the population [17]. Like many other developing countries, Pakistan is also struggling to overcome HIV infection rates due to the use of unhygienic equipment in healthcare departments, an unstable economy, and a lack of education on safe sexual practices [18]. According to a study done by Patel et al. [19], it was concluded that many early cases of HIV went unrecorded with rapid testing, and additional NAAT testing was able to detect many cases of HIV in the window period. This reinforces the idea that better screening and good oversight are required and the need for NAAT to ensure cases of transmission via blood are limited.

While serology testing can filter out most cases of HBV, HCV, and HIV in blood donors, NAAT offers a much higher sensitivity for detecting viral illnesses. Relevant to our study of HIV, HBV, and HCV, the CDC has highlighted the screening methods to follow for each of the viruses. For HBV, HBV surface antigen assay, total antibody core antigen assay for HBV, and NAAT for HBV must be employed. For HCV, an assay for HCV antibody and NAAT for HCV should be done. For HIV, an assay for HIV type I and II and NAAT for HIV type I should be conducted [20]. At our department, these screening methods were followed accordingly to confirm the prevalence of each virus in our blood donor products. Currently, serology testing for HBV, HCV, and HIV is considered mandatory for all blood donor screening, while NAAT is encouraged but not mandatory [3].

While the exact number of blood banks using NAAT to screen blood products is unknown, some studies have reported that only a few transfusion centers are able to provide this service [3,21]. A 2011 study surveyed 17 blood banks in Karachi, Pakistan, and collected data on safety practices, including screening for transfusion-transmissible infections [21]. Regarding HIV, HBV, and HCV, all blood banks performed serological screening via rapid tests or semi-automated enzyme immunoassays to detect these viruses. The study reported that blood banks maintained the standard practice of rejecting seropositive donors and sending them for further confirmatory testing at a clinical laboratory.

Most transfusion centers lack NAAT testing due to the higher costs to implement it and prefer to send patients where NAAT testing is available, as previously discussed [21]. While data on the cost-effectiveness of using NAAT to detect HBV and HIV was harder to find, there was tangible data on the cost-effectiveness of NAAT being used for detecting HCV. One study analyzed different methods and their costs for the elimination of HCV in Pakistan [22]. In accordance with local rates and including staff costs, each antibody screening test was estimated to be around $10-17, while each RNA test was estimated to be around $34-41. Another study explains that in order to make HCV NAAT testing resources adequately available in Pakistan, an investment of US$1.32-1.6 billion would be needed, and by 2030 it would be very cost-effective, saving 29 US dollars per disability-adjusted life year [16]. Moreover, by 2031, the investment would be cost-saving, resulting in an economic benefit of approximately US$9.1 billion in 2050. The study also explains that scaling up on treatment and screening in order to eliminate HCV in Pakistan would avoid 5.57 million disability-adjusted life years, which represent morbidity, and would prevent significant mortality, more specifically, 333,000 deaths related to HCV from 2018 to 2031. However, the national hepatitis profile did report that testing and treatment for hepatitis are now free in public, government-owned blood banks in Pakistan, making it feasible for more patients to get tested [14]. Overall, the data on progress in Pakistan toward hepatitis elimination is still lacking due to Pakistan's lacking a centralized national database registry [23]. Over the period of January 2021 to December 2022, while Pakistan’s government contributed 22.9 million and the Global Fund gave $41.1 million toward the budget for fighting HIV, only $37 million was used for education, testing, and treatment [24]. While data on costs and expenses solely for NAAT were hard to find, the United Nations Aids organization reported that HIV testing and antiretroviral medication for treatment are also free for patients under government-funded facilities. However, Pakistan still continues to have one of the lowest testing and treatment coverages in Asia for HIV, specifically with the main communities affected, testing coverage is 6% in men who have sex with men, 15% in male sex workers, 30% for transgender sex workers, 6% for female sex workers, and 17% for persons using injectable drugs.

NAAT testing is important in detecting the window period of viral infections. This helps in preventing transfusion-transmitted infections after serology testing. Failure to detect HBV, HCV, and HIV in blood donors leads to increased morbidity and mortality rates, which increases the burden on the healthcare system, as compared to other countries [12]. In Pakistan, there is a higher risk of transmission for transfusion-transmitted infections due to inadequate screening [23]. Our study showed that the use of NAAT to detect the window period of HBV, HCV, and HIV is beneficial in reducing the rates of transfusion-related viruses. In our study, 93.02% (n = 43,215) of donors who were seronegative were re-screened for viral infections using NAAT, and an additional 19 (0.044%) HBV and four (0.009%) HCV were found to be in the window period. Had the rescreening using NAAT not been done, these cases would have gone unnoticed and may have passed on the virus. Agarwal et al. [25] and Makroo et al. [26] also used NAAT to rescreen HBV detection and observed similar findings in their studies. Dong et al. rescreened 178,447 blood donor samples using NAAT and found 169 HBV cases that were unnoticed in the initial screening for the virus [27]. An additional 19 (0.044%) cases of HBV were found out of 43,215 blood donors when rescreened with NAAT, while there was a lower NAAT HCV yield rate in the study done by Ali et al. [5] and Hans et al. [28]. In our study, there was no NAAT HIV yield, similar to Nübling et al. [29], who reported a lower NAAT HIV yield rate in their study, and in line with Dong et al. [27], who detected no NAAT HIV yield. A study conducted in Mozambique reported that donor blood that was serologically negative for HBV, HCV, and HIV had higher frequencies of the virus after being analyzed by NAAT [30]. A total of 12.5 HBV cases per 1,000 blood donors, 2.6 HCV cases per 1,000 blood donors, and 2.6 HIV cases per 1,000 blood donors, respectively, were found for the study, contrary to our results [30].

Overall, our study was limited to one center in Karachi, Pakistan. However, our study had a large sample size of 46,455 donors, a sample size similar to other Pakistani studies, and took place in a semi-private hospital, which receives many patients from different parts of Pakistan on a daily basis. Instead of healthy, paid, or volunteer donors, our study consisted of about 90% (n = 41,809) of replacement donors, meaning family or friends of the patients admitted at Dow University Hospital, Ojha Campus, who donated to help them. This contributed to a higher risk of transmitting infection, which is why NAAT was used as a confirmatory test to reduce the risk. Overall, in order to make NAAT a routine screening method in Pakistan, it is important that the concept of measuring NAAT testing’s effectiveness as a confirmatory detection method is further studied. Therefore, large-scale, technical, and cost-effective studies are needed.

## Conclusions

Our study detected the window period of HCV, HBV, and HIV viruses in donor blood with NAAT used as an additional routine test to serology in all seronegative blood donors prior to transfusion at a tertiary care hospital. By documenting this data obtained, we hope to raise awareness about how transfusion-transmitted illnesses can be reduced within the Pakistani healthcare system by simply adding an extra step of NAAT to screen blood products. The importance of safety measures taken during blood transfusions can further reduce the number of viruses transmitted and can continue to lower the burden of these viruses in a country like Pakistan, where the health infrastructure is a struggling one and needs minor adjustments to repair itself majorly. Thus, adding NAAT would be a minor addition to screening that could greatly reduce transfusion-transmitted illness in Pakistan and significantly impact the disease burden. Government policy changes and budget allocation would allow for NAAT to be used in all transfusion centers. As NAAT is already a standard method used to screen blood products in many Western countries, it is even more necessary for Pakistan to use NAAT to reduce its burden of transfusion-transmitted infections. Ultimately, this would bring Pakistan closer to the same level as developed countries in terms of ensuring the safety of donor blood products and their recipients, as well as the level of burden of HBV, HCV, and HIV.
